# Gendered sexual uses of alcohol and associated risks: a qualitative study of Nigerian University students

**DOI:** 10.1186/s12889-016-3163-1

**Published:** 2016-06-06

**Authors:** Emeka W. Dumbili

**Affiliations:** Department of Social Sciences, Media and Communications College of Business, Arts and Social Sciences, Brunel University London, Kingston Lane, Uxbridge, UB8 3PH London, UK

**Keywords:** Alcohol, Alomo Bitters, Aphrodisiac, Gender, Nigeria, Sex, Sexual risk behaviour, Students

## Abstract

**Background:**

Alcohol misuse among young people is a global phenomenon. In many countries, young people engage in heavy drinking and this exacerbates risky sexual behaviour. In Nigeria, alcohol held multiple roles in the traditional era but was mainly consumed by adult males for pleasure. Adult females and young people were culturally constrained from drinking in most communities. In contemporary Nigeria, young people’s drinking is increasing, and many engage in sexual intercourse under the influence of alcohol.

**Methods:**

This study draws on the traditional gender and social sexual scripts to explore the factors that motivate young people to use alcohol for sexual purposes. In-depth interviews were conducted with 19 to 23-year old male and female undergraduate students from a Nigerian university. Thematic analysis was conducted with the aid of NVivo 10 software.

**Results:**

Men drink to become confident to initiate sexual relationships, stimulate sexual urges, prolong erection, increase sexual satisfaction and become more aggressive during sexual intercourse. Women also drink to be bold in initiating sexual relationships, for sexual arousal and to increase satisfaction. Relatedly, not every brand of alcohol is used for sexual purposes. For example, while men use ‘herbal’ alcoholic beverages and a mixture of locally-produced gin and marijuana, women use champagne and other flavoured alcoholic beverages. The results also revealed that young people use alcohol or salt in a bid to prevent conception after sexual intercourse.

**Conclusions:**

Adherence to the traditional gender (masculinity) and social sexual scripts amongst men and the enactment of what appears to be a new form of femininity script amongst women contribute to a culturally specific understanding of the motivations to use alcohol for sexual purposes. Evidence-based strategies should be employed to distribute information about the consequences of sexual intercourse under the influence of alcohol.

## Background

Internationally, research shows that young people engage in hazardous alcohol consumption and this increases the risky sexual behaviour among them [1, 2]. In sub-Saharan Africa, evidence shows that while the number of young people who are involved in heavy episodic drinking is increasing [3], some of these drinkers report engaging in sexual intercourse under the influence of alcohol [4, 5].

In Nigeria, alcohol consumption among young people has been restrained due to socio-cultural beliefs that alcohol is for adults [6], and sexual activity among unmarried youths is also widely taboo [7]. Irrespective of these constraints, young people in contemporary Nigeria, especially students, consume alcohol with a myriad of motives [8, 9]. Again, evidence of sexual activities among unmarried youths abounds [10, 11], and media reports on the use of ‘Alomo Bitters’^1^ and other similar herbal gins as aphrodisiacs are growing [12, 13]. Although a considerable number of studies can be found on alcohol consumption among Nigerian youths, none to my knowledge has examined the motivations to use alcohol for sexual purposes. This study attempts to fill this gap.

### Motivations for alcohol use in sexual situations

Across human societies, many psychoactive substances such as alcohol, cannabis, etc. have been used for sex-related activities for many centuries [14]. Although many psychoactive drugs are used for sexual purposes, alcohol is the most commonly used substance [14]. In fact, Morojele et al. (p222) [15] argued that, among men, a direct link appears to exist between alcohol and sex: users believe that alcohol and sex is ‘a match made in heaven’. Indeed, complex relationships appear to exist between alcohol and sex. For example, while some individuals use alcohol to pay for sexual intercourse [15–17] or to *do gender* in sexual contexts [18], others use drunkenness as a ready-made excuse to engage in unplanned sexual activities [19].

Similarly, Cook et al. [20] revealed that some individuals who use alcohol and other drugs as aphrodisiacs also use them to blur or deaden their psyche in order to forget messages about safe sex. The contexts of alcohol consumption such as bars also facilitate sexual negotiations [21] because drinking together creates opportunities to initiate sexual moves [22] while heavy alcohol consumption is used ‘to become bolder in approaching females and to have lyrics’ (p70) [23].

In a study that investigated the sexual uses of alcohol and other drugs in nine European cities [24], it was found that one-third of the male and a quarter of the female participants used alcohol and other drugs purposefully to achieve sexual prolongation, enhance arousal, increase sensation and derive sexual excitement. Heavy drinking is also perceived to provide boldness in overcoming stigmatisation among men that have sex with men [25], but one serious implication of using alcohol to enhance sexual experiences is that people tend to be so locked into this practice that they rarely give it up [24].

### Consequences of sex under the influence of alcohol

Sexual intercourse under the influence of alcohol has many consequences, and foremost amongst these is regret [26]. Alcohol as a psychoactive drug has amnesic effects that are capable of making users forget the consequences of sex when inebriated [14]. Evidence suggests that young people often engage in unplanned sexual activities [27] with casual partners [28] or are raped [29] under the influence of alcohol. In a study conducted with 9997 Canadian students, it was reported that 68 % of males and 61.5 % of females had engaged in unplanned sexual activities under the influence of alcohol and other drugs [30]. Harmful use of alcohol and other substances results in unprotected sex [7, 15, 31, 32], and this has been reported to increase the chances of contracting HIV and other sexually transmitted infections (STIs) [33].

The use of alcohol (and other drugs) as aphrodisiacs also has other serious health implications such as erectile dysfunction [34], pain during sex, a lack of vaginal lubrication and the inability to attain orgasm [35, 36]. Additionally, although individuals presumably use alcohol to prolong sexual intercourse, a prolonged erection beyond its natural limit causes injuries due to reduced lubrication and facilitates the contraction of STIs [37]. Irrespective of these and other risks, in Nigeria, alcohol can be regarded as the contemporary aphrodisiac among young people [12].

This study presents the findings of a wider study conducted among undergraduate students from a south-eastern Nigerian university. The data sets have been drawn upon to produce a work that focuses on the role of alcohol in identity construction [38]. The current paper explores three interrelated research questions: (I) what are the motivating factors to use alcohol for sexual purposes among undergraduate students? (II) Are the motives for using alcohol for sexual purposes gendered? (III) Where are the motives for using alcohol for sexual purposes learned?

## Methods

### Procedure and participants

This study was conducted on a university located in a city of Anambra State, south-eastern Nigeria between September and December 2013. Thirty-one (22 males and 9 females, aged 19–23 years) undergraduate students were included in the study. All but one of the participants was from the Igbo^2^ ethnic group, and all self-identified as Christians. The participants were recruited across the nine faculties on the university campus using word-of-mouth and snowballing techniques. On campus, the researcher approached students and introduced the project to them. After establishing a rapport, the students were then asked if they drank alcohol. Those who self-identified as current alcohol users were then asked if they would consider participating in the study in order to share their experiences of alcohol use. Those who indicated an interest were provided with an information sheet that detailed the aims of the study, the role of the participants, the potential benefits and harms of participation, the methods for securing data and maintaining confidentiality, and the voluntary nature of participation.

Whilst 26 (20 males and six females) were recruited via this word-of-mouth method, snowballing techniques facilitated the recruitment of an additional three females and two males. These methods were required for the successful recruitment of female participants. While alcohol consumption amongst young people is a sensitive topic in Nigeria and elicits sociocultural disapproval, young female drinkers are particularly stigmatised. Young people, especially females, are not easily accessible for such studies, and reaching them through any means that may expose their identity will hinder their participation. No incentive was given to the participants because the ethical procedure that guided this research did not support it. While the names used in the results section are not the participants’ real names, I adhered to the BioMed Central editorial policies for reporting qualitative studies.

### The interviews

Thirty-one in-depth interviews lasting 33–90 min were conducted. All of the interviews were conducted in the English language by the author (a male), and the participants were fluent. The interviews were recorded with a digital device with the permission of the participants. After testing the interview protocol in a pilot study, an interview guide was developed. While this allowed the interviewer to properly manage the interview sessions, it allowed for unexpected findings to be elicited [39]. The protocol consisted of 12 main questions that were expanded through probes during the interviews.

Some of the specific questions that were asked included: ‘anytime you hear about alcohol, what comes into your mind?’ Can you tell me why you use alcohol?’ ‘What brand of alcohol do you use?’ ‘Why do you prefer that brand?’ ‘Can you tell me the quantity you normally drink on an occasion?’ Can you share some of the experiences you had under the influence since you started drinking alcohol? Those who disclosed sex-related experiences/uses of alcohol were asked: ‘can you shed more light on why you drink to enjoy/prolong sex?’ ‘Who among males and females engages in this practice?’ ‘Can you explain why they do that?’ ‘Where do you think this practice is learned?’ ‘Can you share with me how you learned to use alcohol for sex? This facilitated the exploration of the extent to which they use alcohol for sexual practices and where they learned these practices.

### Data analysis

The interviews were transcribed verbatim and a thematic analysis was undertaken [40]. The data analysis drew upon the traditional gender script [41, 42], which suggests that men and women are socialised into different gender roles that in turn frame their attitudes and behaviours toward sexual activities. Men’s socialisation for instance frames ‘sexual activity as normal for males’ (p271) [41], and enacts abstinence as unmasculine [43]. Women by contrast, are socialised to view sex differently: they are to be passive and act as gate keepers [44]. Among the Igbo ethnic group, abstinence before marriage is culturally approved. In the same culture, the masculine gender scripts equate maleness with being sexually virile and stipulate that men should be daring [41]. This study explores how the participants negotiate and manage this tension in sexual contexts.

As Silverman [45] suggested, a preliminary analysis was initiated immediately after the first interview. The field notes and audio recordings were reviewed to check for accuracy and to identify additional areas to explore further in subsequent interviews. Tentative coding schemes were developed at an early stage [40] with initial extracts categorised into broad themes and subthemes. This provided an early grasp of the data [46], and some of these subthemes, which were grouped manually, became the parent nodes while others were condensed [47] into different child nodes that formed the thematic coding framework when the data were imported into the NVivo software. When the 31 interviews had been completed, each transcript was read many times in order to identify patterns in the data sets [40]. To guarantee consistency and coherence, this process was repeated a number of times before coding was completed. Collaborative analysis between the author and two academic supervisors was also adopted to ensure analytical rigour [48]. As such, themes and subthemes (e.g., “doing gender in sexual contexts”; “aspirations and desires”; “brand preferences for sex”; “drinking to lower inhibition” and “drinking to prevent conception”) were identified.

## Results

### Drinking alcohol for sexual purposes

#### Women’s aspirations and desires: pleasure and drinking to lower inhibition

The participants stated that while some of them (or their friends) intentionally drink in preparation for sexual activities, a few engage in sex spontaneously under the influence of alcohol. To exemplify how alcohol is used before sexual activities, I will largely draw on Agatha’s account. Her account is worth reflecting upon in some detail because it is a useful exemplar of how alcohol is intentionally used for sexual purposes, especially in preparation for sexual intercourse among females. As with the other participants, I started by asking her what comes into her mind when she hears about alcohol, her drinking pattern, those of her friends and what motivates her to use alcohol. Like many other participants, Agatha repeatedly argued that she is “not an alcohol person” (i.e., she does not drink too much alcohol), but noted that she drinks when she wants to do “extraordinary things”. When I asked her to shed more light on what she meant by drinking to do *extraordinary things*, she said:

[Laughs] It’s funny… kind of; let me say sexual life. There is a kind of excitement you get when you take alcohol, and you wanna have sex… ‘I know that when I take alcohol, it puts me in the mood, it makes me feel horny’; it makes me feel like I wanna have sex… And at that moment I am happy… The way I feel when I take alcohol is not the way I feel when I don’t take alcohol. When I don’t take alcohol and I wanna have sex, I just have [sober?] sex but when I take alcohol and I have sex, it is as if it is extended… It is hard to get satisfied at that moment and you want more; you just want more of it…and you enjoy it more too (Agatha, 21).

Other interesting aspects of Agatha’s account are that not every type of alcohol would serve a sexual purpose, and there is a particular quantity that must be used before sex. She shed light on how she tasted different types of alcoholic beverages and thereafter argued that ‘champagne’ is the only beverage that makes her feel “horny” and the quantity she drinks before sex is a whole bottle. When she was asked to unpack how she learnt to use alcohol, particularly champagne for sex, she noted:

Okay, it is out of experience; one day I took alcohol and I noticed that I felt different while having sex and I tried it again and I then noticed that that particular alcohol [champagne] does such a thing to me (Agatha, 21).

Because Agatha mentioned that she has a boyfriend, I probed her to find out whether he knew about this practice, and she narrated that she drinks with his knowledge for sexual reasons:

Yeah, both of us know because sometimes he will tell me, ‘baby I want you to take alcohol because when you take alcohol I like the way you act’ …One thing I know about him is that whenever he takes alcohol, he lasts more [longer] …He is my guy so I know that when he doesn’t take alcohol and we have sex, he comes ‘normal’ but whenever he wants to act like I said in an ‘extraordinary way’, whenever he wants to act more so that I will like it more, he takes alcohol… He takes alcohol just to satisfy me very well (Agatha, 21).

Agatha’s perception of her boyfriend suggests that he may be using alcohol to delay “premature” ejaculation, prolong sexual intercourse and in turn satisfy his girlfriend who also drinks in quest for sexual arousal and pleasure. In so doing, he fulfils two purposes: he derives pleasure, and by adhering to traditional sexual scripts in which ‘good’ sex is long-lasting and penetrative, he also maintains superior masculinity. This is because in a patriarchal society like Nigeria, men who are unable to satisfy their female partners do not command their respect (i.e., people cast doubt on their virility).

Agatha’s account seemingly matched Chioma’s- another female who uses alcohol for sex. Chioma prefers ready-to-drink alcoholic beverages and uses two bottles. She argued that alcohol increases her sexual urge: “I have witnessed it… I was really horny, and all that stuff with alcohol [laughs]”. She indicated that although males mainly engage in this practice, some of her female friends also drink for sexual reasons:

There is one of my friends; she was having her birthday party… So she took alcohol…; so she was just touching herself [demonstrating with body movement], feeling very horny [laughs]; that day was funny. There was her [boy]friend… she didn’t even care whether people were around her; all she wanted was someone who would touch her and ‘sleep’ with her… (Chioma, 21).

From Chioma’s account it can be inferred that she perceives her friend as someone who uses alcohol to lower her inhibition in order to attract sexual attention. In doing so, she appears to manoeuvre the inherent social risks of seeking sexual attention in a patriarchal society like Nigeria, but sex under the influence may create health risks for her.

A slightly different picture was painted by six other female participants, in that they used ‘some people’ to describe how alcohol is used for sexual activities and its consequences. Chichi (aged 23), for instance, stated that she uses up to two bottles of red wine on a drinking occasion but added that acting under the influence of alcohol can result in non-consensual or unprotected sex:

People that get high through drinking alcohol might end up having unprotected sex which can lead to sexually transmitted diseases, pregnancy and abortion…. (Chichi, 23).

When I probed her to unpack how alcohol is used for sexual reasons, who among the males and females engage in such a practice and the motivation behind it, she revealed that the practice of using alcohol for sexual purposes was common among males and that the reason is to be more active during sex. Chichi also drew on her friend’s experience to demonstrate that some females engage in such a practice:

…Some of my friends do it; like there is this friend I have… when it comes to sex, she needs to drink alcohol for her to be like [laughs] relaxed to have sex with her boyfriend or do stuff very well. … She has to drink, and when she gets high, she can really be very active [demonstrating with body movement]…(Chichi, 23).

These accounts suggest that alcohol has complex relationships with sexual activities, because while it is intentionally used to increase sexual urge and become sexually active, some drink to experience elongated sex (arguably to attain orgasm) and enjoy sex. It is also clear that women who use alcohol for sexual purposes share their experiences with their friends and this appears to be one of the ways in which others learn.

Additionally, five female participants reported that they were aware that intoxication could lead to unplanned sex or rape while one revealed that some females use alcohol to boost their confidence in order to initiate or negotiate sexual relationships with men:

…I am an example kind of [laughs]. I might be ‘hitting’ on a particular guy; I mean, if I love this guy and maybe we meet in a nightclub or a party; ordinarily walking up to him to tell him that I love him is something I can’t do, but for the fact that I have just taken alcohol, it makes me bold and I can just come, ask him for a dance and from there we get talking, exchange numbers… yeah; basically, it works for both males and females (Patience, 23).

As indicated above, due to the socio-cultural norms in Nigeria, initiating a sexual relationship is taboo for females. Therefore, to overcome this social constraint, it appears that some females use alcohol purposefully either to lower their inhibition, to boost their confidence or as an excuse to initiate sexual relationships.

### Alcohol use and Males’ sexual behaviour

#### Drinking to lower inhibition, stimulates urge and erection

Out of the 22 male participants, only one argued that he does not know about using alcohol for sexual purposes. As with the women, the men also discussed how they use alcohol to lower their inhibition or boost their confidence in order to initiate sexual relationships:

Alcohol increases confidence; we guys know that it gives us a little bit of confidence… Okay, let me use myself as an example… before you approach a girl, you’ll have this little anxiety or fear of how she might react… but when you’re actually ‘high’… you don’t have time to think about such fears and anxiety; you just go straight to her… We guys know that girls like guys that are confident and strong. So with the help of alcohol, most times you actually get a girl (Chike, 21).

Other men provided additional accounts of using alcohol to boost their confidence to negotiate sexual relationships. While Buchi (aged 23) repeatedly said that alcohol “removes that shame that makes you feel that if I go, the girl may say no”, Larry (aged 21) revealed that drinking to approach girls is popular amongst shy students because it helps them “to get more morale”.

Other findings of interest are that alcohol increases sexual urges and stimulates erection:

…I would say that when you drink …it will make you desire to have sex… and I noticed that if you’re drunk and you’re with a girl, normally this is how you feel; as in the urge. You know as a man it increases the urge seriously and at the end of the day you see yourself having sex… (Jacob, 23).

Some people drink alcohol because it helps them to get an erection when they want to have sex with their girlfriends. That’s why they have to take alcohol (Chike, 21).

Although Chike explained that alcohol can facilitate sexual urge, a significant part of his account shows that alcohol also makes users unable to control their actions:

…I just feel it… makes you less able to control your urges… Every guy has a sexual urge once in a while, but on a normal day, you are able to suppress your sexual urge. But when you take alcohol… you become less able to suppress your urges. It is not only sexual urge; it affects most urges… So what alcohol does is just to make your brain very weak and make you unable to suppress some urges, of which sexual urge is one of them (Chike, 21).

From this account, it appears that drinking alcohol may expose the drinker to unplanned sex and the associated risks because the person is unable to suppress the urge and say no to sex at that particular time. Together, these accounts show that diverse views were held on the effects of alcohol on sexual urges and erection, but none of the participants who use alcohol for sex-related purposes was averse to the fact that alcohol increases sexual urge.

#### ‘Doing gender’ in sexual contexts

The men also discussed how they use alcohol to enhance sexual intercourse. They revealed that alcohol improves sexual experiences because it either helps to elongate sexual intercourse or to derive sexual pleasure:

People do it, but it’s not really like maybe when you want to do it [have sex], you rush after alcohol, but you find yourself going out with a girl [on a date] and… when you come back something happens [actual sex]. You just want to tell her that you’re good. Alcohol does it anyway; it works (Peter, 23).

Interviewer: Okay, how about using alcohol to last longer during sex?

That is to enhance it; to make you do better. Definitely… when you take alcohol, that’s when we can do better, and it might just make you be a bit ‘aggressive’ or something like that. So it helps, but it is not what you will be doing every time, it’s occasional… (Peter, 23).

Peter’s account shows that although he may have experienced sex under the influence of alcohol, it usually happens spontaneously. Buchi (a self-confessed sex-related alcohol user) also revealed that alcohol can facilitate prolonged sexual intercourse:

…When you drink alcohol it makes you more active and makes you last longer… it makes you last longer when you perform… (Buchi, 23).

Larry revealed that alcohol helps to elongate sexual intercourse but added that it enhances pleasure too because “if you really wanna have a good time, you have to be drunk”. He also stressed that the use of alcohol to enhance sexual pleasure or to elongate sexual intercourse is popular among Nigerian men. Indeed, these young men reproduced ‘the traditional male sexual script in which sexual activity is goal-oriented and motivated by bodily pleasure’ (p498) [44]. Again, the penis-centred part of this traditional masculinity sexual script is also played out and the motivation appears to be self-centred (i.e., to have good time or to last longer). These findings also shed light on the construction of masculinity as seen in the men’s references to: “you just want to tell the girl that you are good”; “to be a bit aggressive”; “to be more active”, etc. This suggests that young Nigerian men not only use the consumption of large quantities of alcohol to perform * gender* [38], but that they also use alcohol-induced sexual exploits to construct a superior masculine gender identity.

#### Brand preferences for sex

Other insightful views were expressed by the men, who revealed that although alcohol consumption facilitates sexual intercourse, not every type of alcohol can be used for sexual purposes:

…I’ve seen stuff like that…where a guy tends to perform better when it comes to sex… I don’t know…how beer intake tends to help in sexual intercourse, but there is this particular drink… It is called ‘Alomo Bitters’; every guy I’ve met has told me the same thing that it helps in sexual stuff, I mean in sexual intercourse with a girl (Boniface, 21).

…If you take Alomo Bitters, you’ll get high and last longer than you expected. You know it is bitter, and it washes away sugar in your body. So it makes you last longer. For me, I know that when you are high, you don’t do things with your mind… So when you are having sex, you don’t put your mind on what you are doing, so you’ll last longer (Collins, 23).

Similarly, a few of the men revealed that stout is what they use for sexual purposes. Achike (aged 21) shared an interesting account of how he used to drink other brands of beer such as ‘Star’ and ‘Harp’ before he tried stout and discovered that it was good for his system. With regard to how he learned to use stout particularly for sexual reasons, he recalled that it was from his friends, who also engage in this practice. He added that although he presently uses three bottles on a drinking occasion, he initiated this practice with only one bottle. Another insightful part of his account is that even though he uses stout, not every brand of stout can enhance his sexual performance. This is why he prefers ‘Legend stout’ (with 7.5 % ABV).

Additionally, Chikere (aged 23) stated that some youths also use an alcoholic substance called “monkey-tail”. When he was asked to explain what this substance is made of, he revealed that “monkey-tail” is derived from the fermentation of locally-produced gin and marijuana. Although he identified that this may be dangerous to health, the quest for sexual prowess in order to boost masculine pride encourage such practices. Additionally, Fred shared an account that shows that alcohol is mixed with other substances to enhance its efficacy:

Normally, when guys gather around, they talk about stuff. They said that it works when you mix it. For example, if you take ‘Star beer’ and drink ‘Garri’ [fried granulated cassava]; you last longer during sex… (Fred, 22).

Perhaps the most revealing aspect of Fred’s account is that when men gather, they discuss this practice, and this appears to be where others who may not have known about this practice learn. As shown above, this was also revealed by Achike, who learnt how to use alcohol for sexual purposes from a discussion with his friends. An insightful part of their lived experience reveals that the men were concerned with prolonged sexual intercourse and this motivated their use of diverse substances in order to achieve this motive.

Together, these accounts have shown that those who use alcohol for sexual reasons hold divergent views on the roles it performs for them. Whilst some drink intentionally before having sexual intercourse, others engage in sexual activities spontaneously under the influence with casual partners or even with strangers. Chioma, for instance, narrated how her female friend who was drunk boasted that she would go and approach a man in a nightclub. Chioma shed light on how she found her friend in a club toilet having sex with the casual partner. Although some of these participants reported that they had boy or girlfriends, a male participant reported that he had engaged in unplanned sex under the influence of alcohol with a girl he met at a bar.

#### Drinking to prevent conception

The data show that alcohol is also used for contraception. The majority of the male participants and all of the female participants knew about the use of alcohol, especially stout, for medicinal purposes (to ease pain during menstrual cycles), but a substantial number of the participants revealed that alcohol also serves as a contraceptive. For instance, Chioma revealed that some females mistakenly think that alcohol will prevent pregnancy:

My friend told me that… if she has sex and maybe her boyfriend releases inside her, that he gives her stout and ‘dry gin’ mixed together and she drinks it. She said it helps her according to what she told me [to avoid conception] (Chioma, 21).

She revealed that females believe that not every type of alcohol will prevent conception. Like Chioma, who indicated that her friend uses a mixture of stout and gin, Agatha also recalled that she knows about the use of spirits:

…I can remember spirits… I have not done it because I have protected sex, but I have heard that when a guy releases inside you and you take [a locally-made] spirit, it flushes the sperm inside you… (Agatha, 21).

Jacob also revealed an interesting account by claiming that although alcohol works for girls who do not want to conceive, they must use it immediately after sex. Although he did not indicate the brand of alcohol that females mistakenly think can prevent conception, he highlighted that using a small quantity will not be effective. He stated that a large amount of alcohol should be used immediately after sex. He also reported that this practice is popular among students: “when you think of contraceptives… apart from drugs, that’s another thing most students think of and that’s what they use”. He added that students also use salt. When I asked where this was learned, he replied:

When you see yourself in this situation [faced with the likelihood of your girlfriend becoming pregnant], you want to ask one or two questions and get to know about this (Jacob, 23).

In these accounts, the participants shared their beliefs about the multifaceted sexual uses of alcohol to demonstrate their lived experience and that of their friends. As the participants revealed that they or their friends use alcohol for different sexual purposes, it is clear that young Nigerians on this campus engage in sex under the influence of alcohol and this creates diverse levels of risks.

## Discussion

This study reveals the gendered multiple sex-related roles that alcohol performs for the participants. The findings show that strong associations exist between intentional alcohol consumption and sexual intercourse among the male and female participants. The lived experience of the participants shows the ways in which they use alcohol to lower inhibition and to boost their confidence to initiate sexual relationships. These findings support the findings of Reid et al. [23], who reported that alcohol is purposely used to boost confidence in order to negotiate sexual relationships. Relatedly, drinking contexts (e.g., bars, nightclubs, etc.) also serve as venues for such negotiations [17, 22]. Traditional sexual scripts stipulate that men are supposed to be sexually assertive (to avoid their masculinity being questioned) and women are to be passive and act as good sexual gate keepers (otherwise their femininity will be in doubt) [41]. The findings show that to escape being labelled a transgressor, men use alcohol to lower inhibition to initiate sexual relationships while women also use alcohol as an excuse to go beyond traditional social boundaries. Here, the masculinity scripts that stipulate that men should be daring and good risk-takers are reproduced while women appear to be enacting a novel form of femininity (by initiating sexual relationships).

It was also found that the essence of drinking alcohol before engaging in sexual activities is not just for libidinal efficacy but also for pleasurable sex. This is in keeping with Kahler et al.’s [49] findings that alcohol is intentionally used for the purpose of enjoying sex. While women drink to arouse sexual urges (i.e., to feel ‘horny’) and prepare for sexual intercourse, men also drink for similar purposes. Again, these findings confirm that the use of alcohol as an aphrodisiac is widespread [15]. Relatedly, alcohol is used to prolong sexual intercourse by both genders. The women used alcohol presumably to prolong sexual intercourse, and this arguably is for the purpose of deriving more satisfaction or attaining orgasm. Different studies [50, 51] have shown that orgasm is the desired climax of sexual intercourse or pleasure, and a lack of it is regarded as a sexual disorder [52]. Again, evidence shows that males experience orgasm quicker than females and one of the reasons why females do not experience orgasm in most heterosexual sex is due to the lack of foreplay or as a result of quick ejaculation by men [50]. Because women use alcohol to prolong sexual intercourse, it can be inferred that they do it in a quest for sexual satisfaction or orgasm.

Indeed, a belief system that tends to perpetuate certain norms about sex and sexuality shows that when men engage in foreplay with women and orally or manually pleasure them, women are more likely to attain orgasm and derive more satisfaction [53]. As Waite and Joyner (p248) [54] argued, ‘sex with the partner who knows what one likes and how to provide it is bound to be more satisfying than sex with a partner who lacks such skills’. This was manifested in the ways in which women drank alcohol with the knowledge of their boyfriends in sexual contexts in order to satisfy each other. As such, these participants reproduced the social sexual script that stipulates how an individual should behave towards a sexual partner and defines what he/she should expect from them [44].

Men also drink presumably to elongate sexual intercourse, but this has health implications for both genders. Whilst prolonging erection beyond its natural limit exposes males to injuries due to friction as a result of a lack of or inadequate vaginal lubrication, this can also facilitate the contraction of STIs [24]. Although men use alcohol to prolong sexual activities, interestingly, it is clear that there is another reason for their use of alcohol for sexual purposes: *to be more aggressive or active during sex*. This and other related findings show that men use alcohol to perform masculinity in sexual contexts in order to demonstrate to their female sexual partners that they are virile.

In Nigeria, the formal and informal social structures stipulate that young people should abstain from sexual activities until they marry [41]. Although this is normative, the inherent values of the informal social structure create tension among young men. This is because masculinity scripts stipulate that men should be sexually assertive, and a deviation from this creates social risks and raises questions about a man’s virility [41, 44]. In fact, Izugbara [41] averred that adherence to these masculinity scripts motivates Igbo youths to use sexual exploits to construct gender identity and gain social capital among their peers. Faced with this dilemma, men choose to use alcohol and other substances to enhance their sexual prowess and avoid male-gendered specific social risks. This finding contributes to a better understanding of how young men use alcohol to enhance the acquisition of this capital, by demonstrating how they drink to manoeuvre around diverse sexual challenges.

It is believed that alcohol acquires specific meaning within particular contexts, and this has been revealed in this study. Despite the fact that some studies concerning Western countries have highlighted the positive sexual expectancies of alcohol [39, 55], the predominant public notion is that alcohol hinders pleasurable sexual activities (i.e., causes impotence, lethargy, etc.). As this current study reveals, the meaning of alcohol in Nigeria (where it is presumed to prolong sexual activity and/or is used to perform masculinity in sexual contexts) appears to be slightly different.

Sumnall et al. [56] reported that sexual intercourse occurred circumstantially after drinking, and this was also revealed in this study. Evidence shows that alcohol use before sexual intercourse can lead to the contraction of HIV or other STIs, due to sex with casual partners and no or inconsistent use of contraceptives such as condoms [57]. Because the participants discussed how they (or their friends) had engaged in sex with casual partners under the influence of alcohol, the risks of contracting HIV and other STIs are increased [33].

Interestingly, brand preference for sexual reasons also differed among men and women and this also reflected the traditional gender scripts in Nigeria. Patriarchal norms in Nigeria confer on men the right to drink beer, gin and other similar alcoholic beverages while women use flavoured beverages [38]. Although some women are beginning to question some of these beliefs [38], this result shows the resilient nature of these norms in contemporary Nigeria.

Importantly, it is clear that the males and females in this current study use large quantities of alcohol for sexual purposes. While females might consume a bottle of champagne or two bottles of wine, some males use up to three bottles of stout while others may use gin. The use of large quantities of alcohol for sexual experiences has many associated risks. Although standard drinks are not specified in Nigeria due to a lack of written alcohol control policies [58], it can be inferred that these youths engage in binge drinking that has severe health consequences, especially because some of these alcoholic beverages contain high ABV. For instance, Alomo Bitters contain 42 % ABV; gin contains 20-43 % ABV, while stout contains 7.5 %. Similarly, one of the serious health implications of using herbal gins is that they contain toxic substances according to a recent laboratory test analysis conducted in Nigeria. According to a media report on the analysis, heavy lead metal was found in the blood of users [59].

One of the unexpected findings of this study was that it uncovered some reproductive health mythologies associated with alcohol use. This is because of the belief that alcohol or salt can prevent conception. The fact that young people use diverse substances as contraceptives suggests that they engage in sex without the use of condoms. As such, this not only increases the risk of unplanned pregnancy, but also heightens the possibilities of contracting HIV and other STIs as well as increasing the gender-specific social risk for the women who are directly involved. In Igbo culture, pregnancy out of wedlock is taboo, and stigmatisation is widespread, especially against females. Some families even go as far as to disown their children if they become pregnant before marriage [60]. With abortion being illegal, it appears that youths often resort to trial and error by mistakenly thinking that these substances will prevent conception.

This practice of drinking alcohol to prevent pregnancy has female gender-specific health risks. Because this is dependent upon the consumption of large quantities of alcohol, it can lead to serious alcohol-related problems due to heavy episodic drinking. For instance, locally-produced spirits (that are mainly produced in unhygienic conditions) contain over 20 % ABV [61], and it was revealed that females use these beverages. Again, users may be exposed to other health problems due to the consumption of mixtures of gin and stout or salt. According to Wiederman (p496) [44], social scripting theory is rooted in the ‘assumption that people learn scripts as a function of being raised in a particular culture’. One of the ways in which individuals learn these scripts is by observing other members of the same culture perform these scripts [44]. While some of the participants learned to use alcohol in sexual contexts through trial and error, others learned through friendship networks.

### Limitations of the study

The main limitation of this study concerns the researcher’s gender. As a male, discussing sensitive issues with females in a complex society like Nigeria is a difficult task. A female interviewer could not be used due to the ethical procedure that guided this study. Similarly, only nine females were included in the study due to the recruitment problems detailed above, although as a qualitative study I did not aim to elicit data that are generalizable to the Nigerian student population as a whole. Another shortcoming is that the data were elicited from one university campus; thus, the views expressed by the participants may not necessarily represent the opinions of other Nigerian students. Despite these limitations, the study has, for the first time in Nigeria, employed a qualitative methodology that is underutilised by Nigerian substance researchers [62] to study an understudied group. In doing this, it extends the existing literature on the motivations to use alcohol for sexual purposes and the associated risks.

## Conclusion

This study has demonstrated that adherence to the traditional gender and social sexual scripts, motivates young Nigerians to use alcohol for sexual purposes. Here, males reproduced sexual scripts based on hegemonic masculinity (i.e., men should be domineering, aggressive, risk-takers, etc.). While some women framed their sexual behaviours on the traditional social sexual scripts, others appear to enact a new form of femininity, which to an extent deviates from the traditional norms of passive femininity, but this also facilitates sexual intercourse that may have health risks.

Studies involving female researchers are needed in order to elicit data with greater nuances on the use of alcohol for sexual purposes among females in Nigeria. It is also imperative to further investigate the motivation to use alcohol (especially herbal gin) for sexual purposes in other universities in all regions of Nigeria, especially now that media reports on the use of herbal gins as aphrodisiacs are growing. The findings also suggest an urgent need for the formulation of evidence-based alcohol control policies that will provide guidelines that can inform alcohol users of the risks associated with heavy alcohol consumption. Also, evaluated educational campaign strategies should be employed to spread information about the risks associated with sexual intercourse under the influence of alcohol and the mistaken use of alcohol as a contraceptive. This will help to reduce alcohol-related sexual risk behaviour among youths.

## Abbreviations

ABV, alcohol by volume; STIs, sexually transmitted infections

## Footnotes

1 Alomo Bitters is a herbal alcoholic gin imported from Ghana; it comes in 750-millilitre bottles and contains 42 % alcohol by volume (ABV).

2 Igbo ethnic group is one of the three major ethnic groups in Nigeria. They live in the south-eastern part of Nigeria where this study was conducted.
